# The Function of the Dentist in Race Betterment*Read before the National Conference on Race Betterment, at Battle Creek, Michigan, January 8-12, 1914.

**Published:** 1914-05-15

**Authors:** C. N. Johnson

**Affiliations:** Chicago, Ills.


### THE FUNCTION OF THE DENTIST IN RACE BETTERMENT.*

BY C. N. JOHNSON, M.A., L.D.S., D.D.S., CHICAGO, ILLS.

A consideration of this subjest calls for a study of the significance of the teeth and mouth as factors in individual and community health. We are rapidly learning the lesson that to better the race we must better the individual, and if we are to better the individual we must add to his physical, mental and moral efficiency. It has long been recognized in a general way that the condition of the teeth has much to do with the health of the individual, but not till recently has the direct relationship between oral hygiene and bodily health been definitely and undeniably traced. We all acknowledge that a poorly nourished body must result in inefficiency, but we have not always studied with sufficient care all of

*Read before the National Conference on Race Betterment, at Battle Creek, Michigan, January 8-12, 1914.

the causes or all of the results of faulty nourishment. This question concerns us most in growing children—not in the growing children of the well-to-do perhaps, so much as those of the great mass of humanity who today are everywhere—particularly in our large cities—being gradually assimilated into our future citizenship.

Let one of these growing children be afflicted with decayed and neglected teeth, what is the result? To say nothing of the suffering which frequently follows with its long train of perverted function and incapacity, we have the immediate result of inefficient mastication. Without mastication we cannot have good digestion, without digestion we cannot have assimilation and without assimilation we cannot have nourishment. Many a child is starving for lack of the necessary apparatus with which to properly prepare the food which is placed before him. And the damage is not merely negative—it may become very positive. The child who is illy nourished intuitively develops a craving for stimulants. Observation has demonstrated the fact that these poor children who are suffering from defective teeth and cannot masticate will consume enormous quantities of coffee or tea if they can get it. And it is not fanciful to go one step further. It may seem a far cry from defective teeth to drunkenness, and yet it is a possible and a perfectly logical sequence. We are not giving these children a fair chance in the world for place, preferment or race betterment if we permit them to grow up with faulty mouth conditions.

Not only this but there is a quite unsuspected and a very real danger to the individual and to the community as the result of defective teeth and broken down roots left in the jaws. The inevitable abscesses from these roots discharge large quantities of pus to be taken up in the circulation or carried into the stomach or lungs, creating a constant poison which should no longer be ignored. A general infection of the system sometimes results from an abscess on a single tooth, and it is not unusual to have a life lost from this cause. These decayed cavities in teeth also form an ideal culture place for pathogenic micro-organisms, which are a constant menace to the individual as well as to others, with whom the individual comes in contact. Cook of Chicago demonstrated the tuberculosis bacillus in the roots of pulpless teeth and traced it down through the jaws to the glands of the neck. There is no question that there have been direct tuberculosis infections from this source. One writer has gone far enough to say that 95% of tuberculosis is due directly or indirectly to faulty mouth conditions, that aside from the cases of direct infection from the roots of teeth there are the numberless other cases where the system is rendered susceptible to tuberculosis through inefficient mastication and its consequent train of evils. We all know that the significant thing in tuberculosis is the factor of susceptibility—that practically every individual is exposed to the tubercle bacillus at one time or another on account of its almost universal existence, and that the reason some people escape its ravages is because of their resistance to its encroachment. Let an individual be illy nourished or "rundown" as the phrase is, let the system be impoverished through faulty assimilation so as to develop a lack of tonicity, and the inevitable result is an increased susceptibility to an attack of tuberculosis. The tubercle bacillus seeks a field where the tissues are lowered in tone, and its invasion is usually the result of a lessened resistance through bad air and lack of proper nourishment. Reasoning from this it is not difficult to connect this disease in its incipiency with defective and diseased teeth.

In the public schools of Chicago there was at one time an epidemic of scarlet fever. The health department quarantined every child afflicted with the disease the regulation time, and yet scarlet fever kept spreading. It was noticed that immediately following the return of the quarantined children to school new cases developed among their associates and it was clear that in some manner these children were spreading the disease even after they themselves had long since passed the infective stage. It occurred to the then commissioner of health, Dr. W. A. Evans, that there could be only two ways in which this might happen—the child might carry the germs of scarlet fever indefinitely in the tonsils or in the cavities of decayed teeth. His first order was that no child who had suffered from scarlet fever should be permitted to return to school till all decayed teeth were filled and the mouth made hygienic. Immediately scarlet fever was stamped out of the Chicago schools. Precisely the same thing happened in the public schools of Valparaiso, Indiana. Dr. Nesbit the health commissioner succeeded through a similar regulation in arresting an epidemic of scarlet fever which had persisted for so long a period that it had practically paralyzed the school system of that city.

These instances are only the merest hint of what might be written on the relationship of defective teeth to the community health, but they must suffice for the present occasion, with the passing statement that nowhere in all the realm of medicine is there a more important question than this of oral hygiene or oral sepsis.

If, then, defective teeth are such a prime factor in physical inefficiency it may be well for us to consider briefly the prevalence of this affection. Few people have any conception of the relative number of children who are growing up with bad mouth conditions which prove a handicap to themselves and a menace to the community. In an examination of the teeth of school children in various communities it has been found that at least 90% of them have decayed teeth. In the public schools of Chicago where nearly 70,000 children have been examined the percentage runs much higher than this. During the month of November, 1913, there were examined 2,231 children, of whom 2,224 were found with defective teeth. When only seven children out of 2,231 in a given community are found with perfect teeth it is surely time that our civic authorities and our boards of health give some heed to this important matter.

In conducting ten free dental infirmaries in the public school buildings of Chicago where the teeth of poor children are cared for we are brought face to face with the appalling enormity of the need of this service. The waiting lists of children seeking relief, and the verdict of the school principals where the infirmaries are in operation are sufficiently striking to impress even the casual observer with the significance of the work. One principal writes:—"We are very enthusiastic over the benefits derived from the work done by the dental dispensary in this school. So far this year, emergency cases and very badly neglected cases have kept the dentist busy every minute of the school day. Needless to say the improved physical condition of these children has helped them to accomplish more in the school room."

Another one says:—"I think there is no question about the need of this dental work in the schools and the good that the service is doing. We find that practically all of the children need attention, and that very few of them have received any. Formerly I had to send many children home with toothache. Now I send none."

This gives me the opportunity of remarking parenthetically that if our school boards would spend one half the amount in a campaign for the amelioration and prevention of disease that they now spend annually for teaching the "repeaters" who are made such by reason of disease it would not only be more humanitarian but it would be an immense saving financially.

Another consideration in this connection having a direct bearing on race betterment relates to the handicap to the boy or girl who is allowed to grow up with deformities of the mouth and face due to irregular teeth. In this age of keen competition for place and preferment the appearance of the individual so far as physiognomy is concerned has much to do with his prospects for advancement. One striking case came under the writer's observation, and it seems worth relating as illustrative of the point under consideration. In one of the eastern schools for girls there is a most estimable woman endowed by nature with the mentality and executive ability to be principal of the school, and to wield a large influence in the educational world. Only one thing prevented her advancement and kept her in a subordinate position. When she was a growing girl some one who had charge of her—let us hope it was not her parents—permitted her to come to womanhood with such an irregularity of her teeth that it was impossible for her to cover her upper anterior teeth with her lip on account of the undue protrusion of the upper incisors. This caused such a deformity of her jaws and face that it detracted immeasurably from the force of character of her countenance, and as one young lady pupil expressed it without quite knowing the reason:—"Somehow you could never imagine her as a principal of a school."

These things give us pause and make us wonder if we have any right to bring children into the world and allow them to grow up with such physical handicaps as shall prevent them from having a fair chance to make their way advantageously in life.

It is to the prevention of disease, the relief of suffering, and the correction of deformities—thus adding to the efficiency and happiness of the individual and the community—that the dentist is committed in his function for the betterment of the race.—Dental Review.

